# Development of 3D Printable Gelatin Methacryloyl/Chondroitin Sulfate/Hyaluronic Acid Hydrogels as Implantable Scaffolds

**DOI:** 10.3390/polym16141958

**Published:** 2024-07-09

**Authors:** Caroline A. Murphy, Aleksandra Serafin, Maurice N. Collins

**Affiliations:** 1 Stokes Laboratories, Bernal Institute, School of Engineering, University of Limerick, V94 T9PX Limerick, Ireland; carolinemurphy56@gmail.com (C.A.M.); aleksandra.serafin@ul.ie (A.S.); 2 Health Research Institute, University of Limerick, V94 T9PX Limerick, Ireland; 3 SFI Centre for Advanced Materials and BioEngineering Research, D02 PN40 Dublin, Ireland

**Keywords:** tissue engineering, biomaterials, ECM, 3D printing

## Abstract

The development of biomaterials tailored for various tissue engineering applications has been increasingly researched in recent years; however, stimulating cells to synthesise the extracellular matrix (ECM) is still a significant challenge. In this study, we investigate the use of ECM-like hydrogel materials composed of Gelatin methacryloyl (GelMA) and glycosaminoglycans (GAG), such as hyaluronic acid (HA) and chondroitin sulphate (CS), to provide a biomimetic environment for tissue repair. These hydrogels are fully characterised in terms of physico-chemical properties, including compression, swelling behaviour, rheological behaviour and via 3D printing trials. Furthermore, porous scaffolds were developed through freeze drying, producing a scaffold morphology that better promotes cell proliferation, as shown by in vitro analysis with fibroblast cells. We show that after cell seeding, freeze-dried hydrogels resulted in significantly greater amounts of DNA by day 7 compared to the GelMA hydrogel. Furthermore, freeze-dried constructs containing HA or HA/CS were found to have a significantly higher metabolic activity than GelMA alone.

## 1. Introduction

Tissue engineering (TE) strategies are emerging as a solution to repair and regenerate damaged tissues [1]. Within the field of TE, the scaffold is of the utmost importance, as it provides the initial structural support upon implantation and provides a three-dimensional template for the synthesis of a new extracellular matrix (ECM). Various scaffolds have been investigated to prompt regeneration, including natural [2,3,4,5], synthetic [6,7] or a combination of both [8,9]. Efforts have been made to provide a structural scaffold with appropriate mechanical properties, such as polyurethane (PU) [10], poly-L-lactic acid (PLLA) [11], polycarbonate urethane (PCU) [12], and polycaprolactone (PCL) [6]. However, in addition to the development of the synthetic structural framework of the scaffold, a natural material should be infiltrated into the framework, to provide cell adhesion sites, signalling cues to guide cell growth and differentiation, and support for nutrient diffusion [13].

Materials that provide favourable biological properties are typically hydrogels [5], which may also be 3D printed with encapsulated cells (bioink), or be acellular (biomaterial ink). For biomaterial inks, cell attachment and proliferation occur within the scaffold as native cells migrate after implantation [14]. Alginate [2,15], decellularized ECM [16], collagen [17], gelatin [13,18] and gelatin methacryloyl (GelMA) [19] are commonly used. As GelMA retains many biological properties, such as cell adhesion domains [20] and enzymatic degradability [21,22], and shows potential in applications such as, for example, cartilage tissue engineering [2,23,24,25], it has been of keen interest in recent years. For example, a study by Machado et al. used marine-derived GelMA for tissue-engineered cartilage repair [25]. However, GelMA does not possess GAGs, which are the major compositional units of the ECM. Therefore, it is logical to include GAGs such as hyaluronic acid (HA) and chondroitin sulphate (CS) [26,27,28]. These GAGs participate in several biological processes [29,30,31,32,33,34], influencing cell proliferation and differentiation [35,36]. For example, CS was combined with GelMA to study the effects of matrix stiffness on mesenchymal stem cells, which showcased that a stiffer hydrogel matrix encouraged superior chondrogenesis [27].

In addition to the development of material compositions, an important consideration is the shelf life of the scaffold to make it commercially viable. Freeze drying is a common technique used to create a product with an extended shelf life. Additionally, freeze drying produces a porous structure that provides a temporary microenvironment for the promotion of cell attachment and proliferation while guiding the formation of new tissue [37,38,39,40,41,42].

Therefore, in this study, we develop a biomaterial ink that largely mimics the composition of the ECM, while enhancing the properties of GelMA-based hydrogels through incorporation of glycosaminoglycans. Following on from this, we assess these materials’ suitability for tissue-engineered applications. To achieve this, the major GAGs found in the ECM tissue, chondroitin sulphate and hyaluronic acid, are incorporated into a GelMA matrix. In the majority of reported studies, methacrylated versions of CS and HA are used as additions to a GelMA matrix [25,27,28]. In this study, however, both CS and HA remain in their non-methacrylated form, thus providing a novel investigation into the combination of these three materials. The structure/property relationships of these biomaterials are investigated in terms of mechanical properties, swelling behaviour, rheological behaviour, and their potential to be 3D printed. An in vitro cell study is conducted to determine cell attachment, cell morphological changes, and proliferation on different ECM-like formulations. Freeze-dried hydrogels were also investigated to determine cell proliferation rates compared to non-freeze-dried equivalents and to increase the shelf life of the scaffolds.

## 2. Materials and Methods

### 2.1. Materials

Gelatin methacryloyl and photoinitiator, lithium phenyl-2,4,6-trimethylbenzoylphosphinate (LAP), were purchased from Allevi (Philadelphia, PA, USA). GelMA was derived from Type A, 300 Bloom, porcine skin and fabricated with a final degree of methacrylation of 50%. GAGs investigated included hyaluronic acid of molecular weight 0.1 MDa (HA) (Shanghai Easier Industrial Development, Shanghai, China) and chondroitin-6-sulfate (CS) (C4384), derived from shark cartilage (Sigma Aldrich, Wicklow, Ireland).

### 2.2. Hydrogel Fabrication

HA and CS were dissolved in PBS overnight at 25 °C. LAP was dissolved in PBS at 60 °C (final concentration of 0.5% *w*/*v*) [43]. Once LAP was completely dissolved, GelMA was added and allowed to dissolve at 60 °C. Subsequently, the GelMA solution was added to the HA, CS, and HA/CS solutions to reach a final concentration as shown in Table 1. While the solution was still warm, it was transferred to a 10 mL syringe. Solutions were vortexed for 1 min, to ensure uniform mixing. Syringes were then placed upside down into 50 mL centrifuge tubes and centrifuged at 1000 rpm for 3 min to remove air bubbles. All syringes were wrapped in aluminium foil to prevent light-induced crosslinking. Pre-gelled solutions were injected into custom-made Teflon moulds, 6 mm in diameter and 2 mm in height. A glass coverslip was placed on top of each mould to ensure uniform samples were created. Hydrogels were produced via crosslinking for 15 min under UV light exposure, wavelength of 365 nm (Model: EA-160/FBE, Spectroline, Westbury, NY, USA). Samples were methodically investigated to determine optimum concentrations of GAG to produce a fully crosslinked gel.

### 2.3. Mechanical Properties

The mechanical behaviour of hydrogels was characterised utilising unconfined compression, using non-porous platens. Stress–strain measurements were obtained using a Tinius Olsen H25KS (Philadelphia, PA, USA), equipped with a 100 N load cell. Once the sample was crosslinked, it was placed on the lower platen of the compression tester. The upper platen was lowered to just above the sample, and subsequently, a 0.1 N load was applied to ensure full contact with the sample. The hydrogel disks were tested at a strain rate of 0.5 mm/min, until 5 N was reached. Compressive moduli were calculated from the linear region of the stress–strain curve.

### 2.4. Swelling Behaviour

Crosslinked hydrogel samples were initially dried in a vacuum oven overnight. The samples were weighed (n = 3) and subsequently immersed in PBS at 37 °C. After 24 h, the samples were removed from PBS and blotted using a Kimwipe to remove any residual liquid, and the samples were weighed. The swelling ratio was determined using the following equation:

$$SR=\frac{W_{w}-W_{I}}{W_{I}}$$

where SR is the swelling ratio, W_w_ is the wet weight after 24 h, and W_I_ is the initial dry weight.

### 2.5. Fourier-Transform Infrared Spectroscopy (FTIR)

FTIR was utilised to determine the chemical interactions upon addition of CS and HA to the GelMA hydrogel. The following groups were analysed: GelMA, GelMA/CS, GelMA/HA and GelMA/CS/HA. Samples were prepared by pipetting 300 µL of each group (n = 3) into a 24-well plate, followed by crosslinking under UV for 15 min. All samples were dried in an oven overnight at 30 °C to achieve a film. FTIR spectra were obtained using a Perkin Elmer FTIR spectrometer (Spectrum 100, Worcester, MA, USA). A background scan was first performed, which was automatically subtracted from the subsequent sample spectrum, to remove any unwanted residual peaks. Scans were performed with a resolution of 2 cm^−1^ in the range of 4000–650 cm^−1^ with a scan number of 10.

### 2.6. Rheological Properties

Rheological investigations of ink samples were conducted on a rheometer (TA Instrument, Discovery HR-2, London, UK) using a plate-plate geometry (diameter of 25 mm, gap of 0.3 mm). The viscosity was assessed using shear sweep analysis at a constant temperature of 25 °C, with a logarithmic increase in shear rate from 1 to 1000 s^−1^ (n = 3). To measure the frequency-dependent storage and loss moduli of the various concentrations, a plate-plate geometry (diameter of 8 mm, gap of 0.5 mm) was utilised. Oscillation frequency analysis was conducted in the range of 0.1–10 Hz at 1% strain. The strain was determined to be in the linear viscoelastic region by performing a strain sweep analysis at a constant 25 °C, with a logarithmic increase in strain from 0.1 to 100%. All samples were held at 25 °C for 3 min prior to test commencement. All measurements were conducted in triplicate.

### 2.7. Printability

To assess printability of the gels, samples were printed using a pneumatic-based Biobots bioprinter (Allevi, Philadelphia, PA, USA). Samples were loaded into a syringe and deposited onto a glass slide using a pressure of 3.8–8.8 psi and a print speed of 2–4 mms^−1^, using a 27 Ga needle. Once samples were printed, they were crosslinked using UV light for 15 min. The filament spreading ratio was determined, whereby the printed line width is divided by the needle diameter as previously described [44].

### 2.8. Freeze Drying

To obtain freeze-dried scaffolds, crosslinked hydrogels were frozen and stored at −80 °C. Samples were transferred to a freeze dryer (Severn—LS40/60, Coventry, UK), and a standard freeze-drying procedure was used whereby the temperature was ramped down from room temperature to a freezing temperature (−30 °C at 1 °C/min. Samples were held for 1 h before being brought back up to a drying temperature of −10 °C and held for 12 h under vacuum. Once freeze-dried, samples were kept sealed in a Petri dish and stored at room temperature until cell seeding.

### 2.9. Morphological Analysis

To create samples for morphological analysis, each hydrogel precursor composition was injected into a 6 × 2 mm Teflon mould, a glass cover slip was placed on top, and crosslinking was performed under UV light at a wavelength of 365 nm (Model: EA-160/FBE, Spectroline, Westbury, NY, USA) for 15 min. Samples were subsequently freeze-dried as previously outlined. Constructs were cross-sectioned using a scalpel, sputter coated with gold (Emitech, k550, Montigny Le Bretonneux, France), and imaged using scanning electron microscopy (SEM) (Hitachi, TM-1000, Tokyo, Japan). SEM images were analysed using ImageJ software (Java 8), and 10 pores were averaged to determine the pore size.

### 2.10. GAG Retention Analysis

As HA and CS are water-soluble, the retention of these GAGs in a freeze-dried GelMA matrix was investigated. Freeze-dried samples were prepared as previously described. To investigate GAG leaching from the scaffolds, samples were placed in PBS, removed at different time points, digested, and the retaining GAG content was quantified. For the CS retention study, constructs were digested with papain (0.1 mg/mL, pH 6.4) in a sodium phosphate buffer containing 0.1 M sodium acetate, 5 nM L-cysteine HCl, and 0.05 M ethylenediaminetetraacetic acid (all Sigma-Aldrich, Wicklow, Ireland) at 60 °C overnight, shaking at 100 RPM. The GAG content was quantified using dimethylmethylene blue dye-binding assay (Blyscan™ Glycosaminoglycan Assay, Biocolor, Carrickfergus, UK) as per manufacturer instructions, followed by measuring the absorbance using a microplate reader (BioTek, Synergy Mx, Vermont, VT, USA) set at 656 nm. Total sGAG was calculated from a chondroitin sulphate standard curve. To identify HA retained in samples, samples were digested in a 50 mM TRIS-HCl buffer containing Proteinase K overnight at 60 °C. The digested samples were analysed using a hyaluronan assay (Purple Jelly Hyaluronan Assay, Biocolor, UK) that was run as per manufacturer instructions, followed by absorption measurements read at 655 nm (BioTek, Synergy Mx, Vermont, VT, USA). Total HA was calculated from the standard curve obtained with the HA standard. Both assays were run at day 0, day 1, day 3 and day 7, and GAG concentrations were converted to percentages retained across time points (n = 3).

### 2.11. In Vitro Cell Investigation

#### 2.11.1. Cell Expansion, Seeding and Culture

Fibroblast cells, L927 (P18), were expanded in culture media DMEM + GlutaMAX (Gibco, Thermofisher, Dublin, Ireland) supplemented with 10% foetal bovine serum (FBS) and 100 units/mL Penicillin and 100 µg/mL Streptomycin (all Gibco, Thermofisher, Dublin, Ireland). Cells were cultured until reaching confluency, changing the medium every 2–3 days. Prior to cell seeding, hydrogel scaffolds and their freeze-dried counterparts were sterilised under UV light for 30 min and soaked in culture medium for 2 h. At the time of seeding, samples were transferred to a new well plate, where well beds were coated with a 1% agarose solution (Sigma Aldrich, Ireland) to prevent cell attachment to the plate. Cells were seeded onto the scaffolds at a density of 150,000 per 30 µL of medium and transferred to an incubator for 30 min to allow for cell attachment. Subsequently, 1 mL of medium was added dropwise. Medium was changed every 2–3 days, and samples were cultured for up to 7 days.

#### 2.11.2. Cell Viability

Live/Dead assay was performed at 3 different time points (day 1, day 3 and day 7) to visualize cell viability and overall morphology. In brief, cell-seeded constructs were washed in PBS and allowed to incubate with Ethidium Homodimer-1 (EthD) and Calcein (both Biotium, Fremont, California, CA, USA) for 1 h at 37 °C. Samples were imaged using a confocal microscope (Zeiss, LSM 710, Germany) at the following excitation/emission wavelengths: Calcein, 495 nm/515 nm; and EthD, 495 nm/635 nm.

#### 2.11.3. Metabolic Activity

Cell metabolic activity was analysed using an Alamar blue assay (Thermofisher, Ireland). Briefly, the medium was removed from each well, and 1 mL of fresh medium containing 10% Alamar blue was added, and incubated for 2 h, followed by reading absorbance with an excitation of 540 nm, emission of 590 nm, and sensitivity set to 100 (BioTek, Synergy Mx, Vermont, VT, USA). Blanks of medium+Alamar Blue were run, and the intensity result was taken from the samples containing cells. Assays were run at day 0, day 3 and day 7 (n = 3).

#### 2.11.4. Cell Proliferation: DNA Assay

To determine cell proliferation, cell-seeded constructs were analysed by quantification of DNA. The sample was first digested in papain extraction reagent and reacted with a Bisbenzimide Hoechst 33,258 working solution (1 mg/mL) as per manufacturer’s instructions. Samples were read using an excitation of 360 nm, emission of 460 nm, and sensitivity was set to 120. Using a standard curve and taking dilution into account, DNA content for each sample was derived. Experiments were performed at day 1, day 3 and day 7 (n = 3).

### 2.12. Statistical Analysis

Significance between sample compositions and time points was determined using a two-way analysis of variance (ANOVA), utilizing GraphPad Prism 8 (GraphPad Software, San Diego, SD, USA), followed by Tukey’s post hoc method to investigate where significant differences occurred. Differences were considered significant at *p*-value < 0.05.

## 3. Results and Discussion

### 3.1. GAG Concentration and Crosslinking Effectiveness

**Figure 1:**
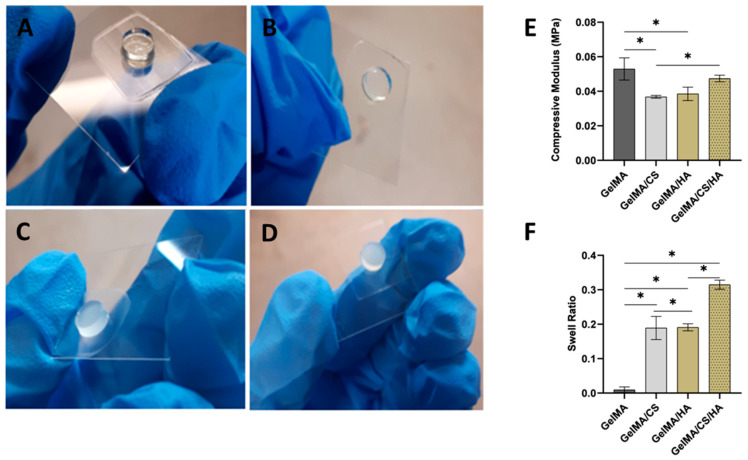
Gelation of 10% *w*/*v* GelMA matrix with the incorporation of different GAGs (**A**) GelMA: 10%, (**B**) GelMA/CS: 10%:1% *w*/*v*, (**C**) GelMA/HA: 10%:0.5% *w*/*v*, (**D**) GelMA/CS/HA: 10%:1:0.5% *w*/*v*. (**E**) Compressive modulus of various biomaterial inks, * depicts significant difference. (**F**) Swelling ratio of various compositions, * depicts significant difference.

Initial GAG concentrations were based on the content of GAGs in the ECM of natural tissues such as the meniscus and in line with the most promising results found in the literature [45]. In previous studies, HA has been found to yield the most promising results between a concentration of 0.5–2% [46,47]. All formulations investigated for crosslinking ability are shown in Figure 1A–D. It was found that the crosslinking capacity of GelMA with HA was concentration-dependent. At a higher concentration of HA at 2 *w*/*v*%, the hydrogel did not crosslink. However, progressively decreasing the concentration to 0.5 *w*/*v*% HA, the hydrogel successfully crosslinked; therefore, this was the concentration of HA used throughout this study. Furthermore, it was found that keeping the concentration constant and increasing the molecular weight of the HA from 0.1 MDa to 0.6 MDa caused insufficient crosslinking of the GelMA hydrogel. It is believed that the incorporation of HA caused phase separation within the GelMA matrix. This phase separation resulted in an inhomogeneous dispersion of HA molecules, which in turn prevented the GelMA chains from crosslinking. This was more prominent as the concentration or molecular weight of HA increased. With higher molecular weight HA, there are fewer chain ends, and this can also result in lower crosslink densities; therefore, higher molecular weights of HA were not further investigated in this study. Phase separation between HA and GelMA was previously observed by Levett et al. [3].

### 3.2. Mechanical Properties

Mechanical properties of hydrogels are well known to be influenced by the crosslinking density of the polymer networks [4,48,49]. The unconfined compression test revealed that the addition of GAGs influences the mechanical properties of the gels. Overall, GelMA/GAG hybrid hydrogels were determined to be mechanically tuneable, with compressive moduli ranging from 36 kPa to 93 kPa. This finding could be useful for several different TE applications. The obtained compressive modulus of GelMA hydrogel was slightly higher but in a range similar to that reported in the literature [20,50,51]. With the addition of unmethacrylated GAGs, there was a noticeable decrease in the mechanical properties, as shown in Figure 1E. This was due to a decrease in the crosslink density as observed by the swell mechanics of the gel. This decrease in crosslink density was due to the GAG molecules, hindering the crosslinking of the GelMA. Interestingly, it was observed that although combining both GAGs caused a significant increase in the swelling ratio, it simultaneously caused an increase in mechanical properties. This was not expected, as a lower crosslink density would be expected to lead to lower mechanical properties. The weak interpenetrating network of hydrogen bonding between the HA and CS as displayed in the FTIR data may explain why the mechanical properties increase even though there is an increase in the swelling ratio.

When comparing the mechanical properties of these hydrogel materials to that of the human tissue, the results are comparable at 60 and 70 kPa for the medial and lateral parts of the human meniscus, respectively, as defined by a confined compression-aggregate modulus by Seitz et al. [52]. Of course, as the meniscus is an anisotropic material, the mechanical properties change depending on the mode and direction of the analysis, for example, radial vs. circumferential direction. The depth of the sample within the meniscus also had a significant impact on the mechanical results, due to the structural variation in the collagen fibres. The superficial zone displayed isotropic properties in both the circumferential and radial plane; however, deeper within the meniscus, the material properties were highly anisotropic. It was reported that the strength is highly dependent on the orientation of the fibres to the tensile axis [53,54,55,56].

### 3.3. Swelling Behaviour

The swelling behaviour of GelMA-based hydrogels was found to be tuneable by varying the composition of the hydrogel components, as shown in Figure 1F. For example, the addition of 1% CS into 10% GelMA caused a large increase in the swelling ratio from 0.01 to 0.19. Similarly, a large increase was observed with the addition of 0.5% HA. This increase in the swelling ratio was due to a decrease in crosslink density, as the GAG molecules hindered the crosslinking of the surrounding GelMA network. There was no significant difference in the swelling ratio between the addition of HA or CS; however, when both GAGs were added simultaneously, there was a large increase in the swelling ratio, to 0.31. This surge in water absorption was attributed to decreasing crosslink densities, as well as to the introduction of ionic groups that increased the hydrophilicity of the hydrogel network. These results further highlight that the incorporation of GAGs into GelMA hydrogels infers the ability to modulate and tune the physiochemical behaviours of these hydrogel systems.

### 3.4. FTIR

**Figure 2:**
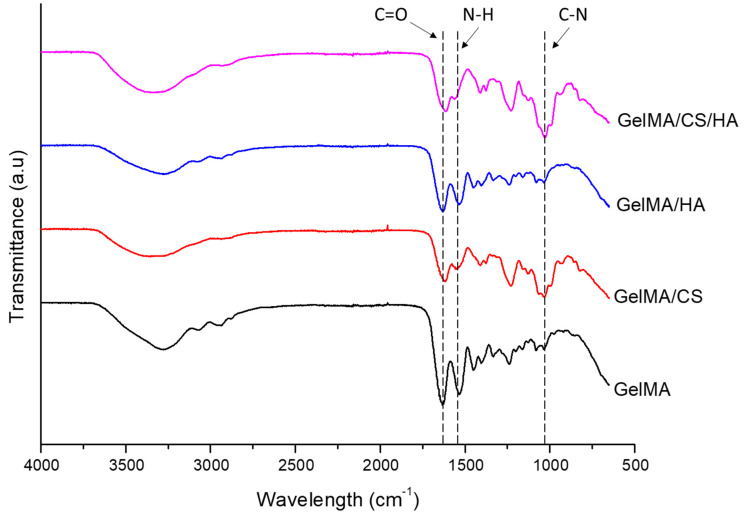
FTIR of gelatin methacryloyl/chondroitin sulfate/hyaluronic acid hydrogels.

To further investigate and determine the interactions occurring within the crosslinked hydrogel network, the assignment and analysis of peaks in FTIR spectra were conducted to identify the changes in the chemical structure upon addition of the studied GAGs, as shown in Figure 2. The spectrum of GelMA was dominated by the vibrations arising from the amide groups and the characteristic bands at 1635 cm^−1^, 1538 cm^−1^, and 1227 cm^−1^ [57,58,59]. These bands predominantly arose from C=O stretching vibrations [58,60,61], N-H bending vibration and C-N stretching vibration [58,61,62] and C-N stretching and N-H in-plane bending, respectively [58].

For samples containing GelMA and CS compared to GelMA alone, some positions of their spectra bands are very close; however, noticeable differences could be seen at 1375 cm^−1^ and 1410 cm^−1^, with coupling of the C–O stretching vibration and O–H variable-angle vibration, indicating the existence of the free carboxyl group [63]. C–O stretching vibration was observed at 1129 cm^−1^ [58], with a peak at 1229 cm^−1^, which was due to the S=O stretching vibration attributed to the negatively charged SO_4_ ^2−^ groups of the CS molecules [60,63]. The broad peak at 1030 cm^−1^ for C-O-C stretching vibration was attributed to the saccharide structure [63,64], while the amide I band of GelMA shifted to a lower wave number of 1616 cm^−1^. This suggests an interaction between gelatin and CS [61]. As the amide I band is sensitive to the change in secondary structure, the shift in band position with the addition of CS suggests the conformational change of GelMA [58]. This may explain the improved moduli reported above.

Very minimal difference was observed in the GelMA spectra when HA was added; this was most likely due to the low concentration at 0.5% [57]. However, a slight movement in the amide I band was observed, lowering to 1630 cm^−1^. This would suggest some interaction between molecules. The addition of HA had more of an impact on the spectra when added to GelMA/CS. The addition of HA to GelMA/CS lowered the amide I band for GelMA/CS/HA to 1613 cm^−1^. The shifting of peak position implies that hydrogen bonding may be occurring between the GAG and GelMA polymers [65]. Furthermore, when both HA and CS were added simultaneously to GelMA, the O-H stretching vibration bandwidth appeared to become broader compared to GelMA/CS or GelMA/HA alone. It is generally accepted that the O-H band width qualitatively reflects the distribution of hydrogen bonds over a variety of binding sites [60]. This may suggest that the addition of the two GAGs simultaneously may have caused increased hydrogen bonding and may explain why the addition of HA and CS leads to a superior crosslinked hydrogel compared to HA alone. Furthermore, this weak interpenetrating network of hydrogen bonding between the HA and CS may explain why the mechanical properties increase even though there is an increase in the swelling ratio.

### 3.5. Rheological and Viscoelastic Properties

**Figure 3:**
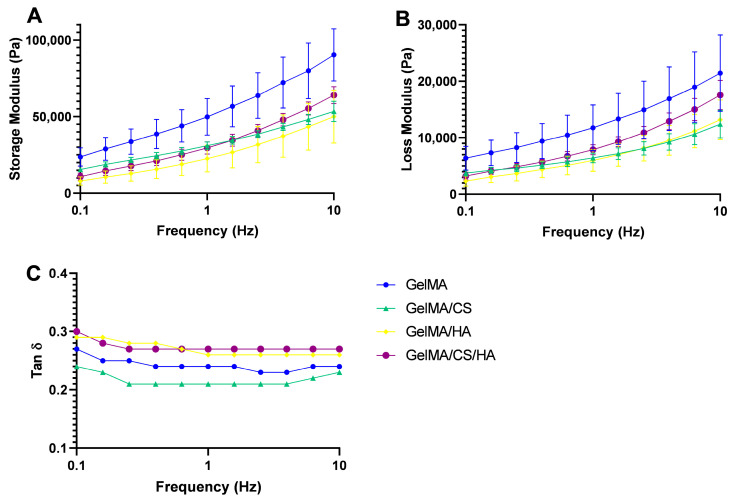
(**A**) Storage modulus, (**B**) loss modulus, and (**C**) tan δ of un-crosslinked biomaterial inks.

To further characterise these molecular interactions and hydrogen bonding described above, the dynamic viscosity of the pre-gelled systems was plotted as a function of the shear rate, as shown in Figure 3. In the range of the shear rate studied (0.01–1000 s^−1^), the rheological properties of the different solutions displayed similar shear-thinning properties. This suggests these materials may be suitable as potential bioinks for extrusion-based bioprinting applications [66,67]. The viscoelastic moduli of all the potential bioinks were frequency-dependent, with both storage (G’) and loss (G″) moduli increasing with frequency. All samples had a tan δ value less than 1, which demonstrates that the storage modulus dominates, and indicates all samples displayed gel-like behaviour at room temperature before crosslinking; this largely concurs with the findings in FTIR, as hydrogen bonding between the GAGs and GelMA gives rise to transient gel-like behaviour [68,69]. Although, the addition of HA alone caused a decrease in storage modulus attributed to its low concentration, the addition of CS caused the storage modulus to increase.

### 3.6. Printability

**Figure 4:**
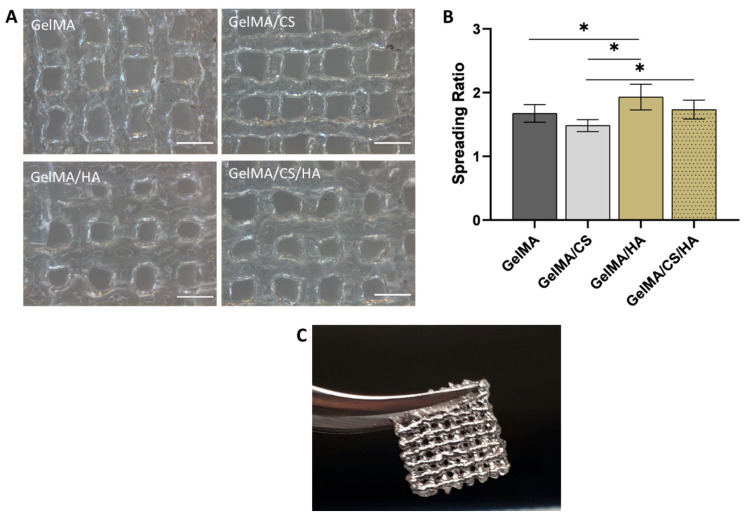
(**A**) Printed ink systems (scale bar represents 1 mm). (**B**) Spreading ratio of printed inks. (**C**) GelMA/CS printed into a stable 3D structure, * depicts significant difference.

The addition of GAGs to GelMA and their influence on printability are assessed in Figure 4. All gels were printable; however, differences in shape fidelity were observed. With the addition of HA, the line spreading was significantly larger than with GelMA alone. When HA and CS were added to the GelMA simultaneously, line stability increased, and the spread ratio significantly decreased. Although the viscosity remained constant with the addition of CS to GelMA/HA, it did, however, cause an increase in the storage modulus. This increase identified that the material had become more elastic and therefore, more energy was stored, which in turn led to more structural stability of printed lines. Overall, from this printability study, it is evident the tan δ is a good indicator of printability for these systems. In Figure 3C, it is evident that at 1 Hz, inks with a tan δ value < 0.24 result in stable prints, while print systems with a tan δ value of >0.24 result in lower structural stability.

### 3.7. In Vitro Viability

**Figure 5:**
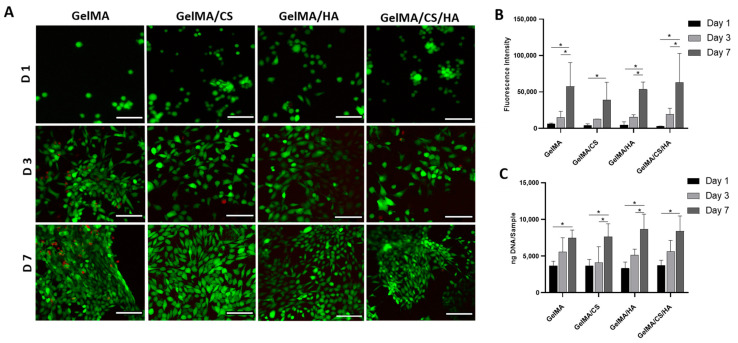
(**A**) Live/Dead confocal images of surface of hydrogels at D1, D3 and D7 (scale bar represents 100 µm). (**B**) Alamar blue fluorescence intensity and (**C**) DNA content of fibroblasts seeded on gel constructs over cell culture in vitro. * depicts significant difference between groups.

Live/Dead images of hydrogel surfaces are shown in Figure 5A, whereby green depicts live cells, with red displaying dead cells. Overall, it was observed that the hydrogels are highly biocompatible, with limited evidence of cell death being observed. It could be observed that the fibroblasts changed their morphology from round at day 1 to elongated fibroblast phenotype by day 7. Cell viability was found not to be group-dependent, with all groups showing high viability for up to 7 days in in vitro culture. Additionally, all constructs were found to facilitate cell growth, as increased cell densities were observed on days 1, 3, and 7.

Furthermore, cell metabolic activity was investigated using an Alamar Blue assay, as shown in Figure 5B. Relative to day 1, all groups showed a significant increase in cell metabolic activity by day 7. Overall, GelMA/CS/HA displayed the highest cell activity at day 7. To identify cell proliferation, the DNA content per construct was analysed. The DNA per construct was found to significantly increase between day 1 and day 7. At day 1, all groups showed similar DNA per construct, indicating that all groups had the same cell attachment. GelMA provides the main biological recognition sites of the arginine-gylcine-aspartic (RGD) sequence, which allows cell adhesion [5,18,70]. All gels contained the same concentration of GelMA and therefore contained the same quantity of adhesion sites from GelMA. Between day 1 and day 3, GelMA and groups containing HA showed the biggest change in DNA content. However, groups containing CS or HA showed the highest cell proliferation between day 3 and day 7. By day 7, groups containing HA and HA/CS showed the highest DNA content, suggesting these groups promote cell proliferation. This increased DNA content may be explained, as it is known that HA and its interactions with specific proteins are implicated in a number of biological processes, such as cell attachment, mitosis, and proliferation [29,71,72,73,74].

### 3.8. Morphological Influence on In Vitro Cell Viability

**Figure 6:**
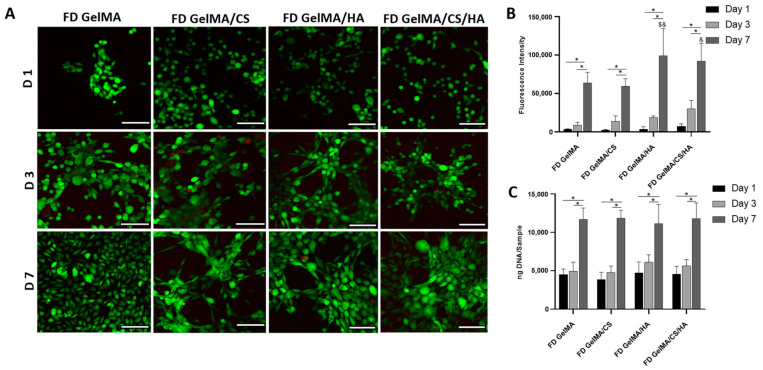
(**A**) Live/Dead confocal images of surface of freeze-dried hydrogels at D1, D3 and D7. Scale bar represents 100 µm. (**B**) Alamar blue fluorescence intensity and (**C**) DNA content of fibroblasts seeded on freeze-dried (FD) gel constructs over time. * depicts significant difference between groups, $ depicts significant difference between GelMa D7, and & depicts significant difference between GelMA/CS D7.

Hydrogels were freeze-dried to determine morphological influences on cell viability and proliferation for these biomaterials. Cells were imaged under a confocal microscope, as shown in Figure 6A. Live/Dead images highlighted that all constructs were biocompatible, with excellent viability observed over the test period. All constructs facilitated cell proliferation, as cell density increased with time. In contrast to the non-freeze-dried hydrogels, freeze-dried constructs of the same composition displayed different cell distributions and cell-to-cell interaction. For example, by day 7, cells followed the shape of the struts on the scaffold. While this occurrence is to be expected [40], cells appeared to be more elongated and formed interconnected networks. This cell distribution and cell-to-cell interaction was particularly evident in constructs containing GAGs compared to GelMA alone, as shown in Figure 6A. Furthermore, cell morphology appeared to vary upon the addition of HA or CS. Samples containing CS appeared to exhibit an elongated structure, while samples containing HA alone exhibited a rounded shape [57].

In Figure 6B, Alamar Blue results show that all studied groups exhibit good cell viability with increasing metabolic activity, both on day 3 and day 7. At day 7, constructs containing HA displayed higher fluorescence than GelMA alone controls, indicating increased metabolic activity with the addition of GAGs. Overall, the introduction of HA appeared to have more of an influence on metabolic activity than variation in morphology from non-porous to porous structures. This suggests material properties have a greater effect on cellular activity than morphology. Between day 1 and day 3, and day 3 and day 7, all groups showed significant increases in DNA content, demonstrating the promise of GelMA-based hydrogels to promote fibroblast proliferation, as shown in Figure 6C.

**Figure 7:**
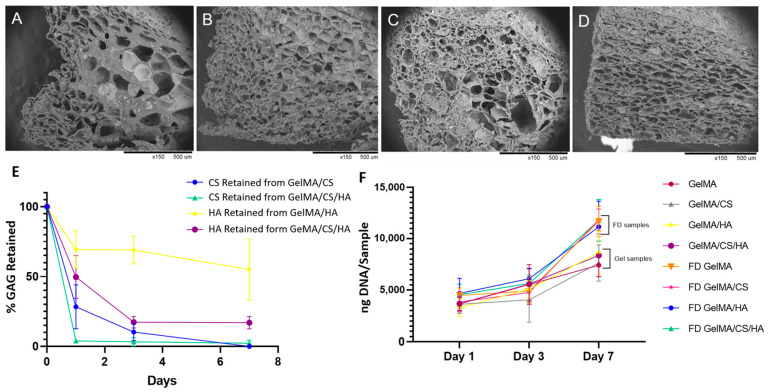
Freeze-dried samples (**A**) GelMA, (**B**) GelMA/CS, (**C**) GelMA/HA, (**D**) GelMA/HA/CS. (**E**) Retention of GAGs in GelMA composite matrix over 7 days, and (**F**) DNA quantification of gel samples vs. freeze-dried (FD) samples. At day 7, all FD samples were significantly greater than GelMA gels.

As shown in Figure 7A–D, freeze-dried GelMA produced a scaffold with larger pores in the centre of the construct and smaller pores towards the periphery. The addition of CS caused a visible reduction in pore size, and a more homogeneous pore distribution could be observed. The incorporation of HA, albeit at lower levels, had less of an influence on pore size compared to CS; however, it also produced a homogeneous interconnected porous structure compared to GelMA alone. Similar results have been observed for HA addition to a collagen scaffold [75].

As Figure 7E shows, HA diffused out of the bulk scaffold over the first 3 days, while some HA remained bound to the scaffold [76]. When HA was incorporated in a GelMA/CS hydrogel, less HA was retained within the scaffold; this may be attributed to a decrease in crosslink density of the GelMA with the addition of two GAGs simultaneously, as discussed. The same trend was observed for CS, whereby more CS diffused out when both CS and HA were added simultaneously. By day 7, only minimal CS (3%) was still retained within the GelMA matrix. Overall, HA was retained within the GelMA more easily than CS, and this may explain why HA has more influence on cell proliferation rates and metabolic activity.

Figure 7F shows that at day 1, the DNA content was higher in the freeze-dried porous constructs compared to the non-porous hydrogels, indicating higher cell attachment. This was due to an increase in surface area, with porous structures displaying a 30–57% increase in DNA content compared to their counterparts at day 7. Overall, these results highlight the advantage of using freeze-dried porous hydrogels containing GAGs over their non-porous counterparts to promote cell proliferation and, by extension, tissue regeneration.

Taking all the analyses of the GelMA/GAG hydrogels into account, these materials are promising for various tissue engineering strategies, for example, meniscal repair. Their clinical applications could encompass the replacement of damaged meniscus and prompt the regeneration of the tissue at the site. However, further research into the in vivo applications has to be conducted to fully support this statement.

## 4. Conclusions

In this work, we investigated GelMA/GAG hydrogels for tissue-engineered regeneration proposes. Firstly, a novel way to incorporate HA at higher molecular weights without phase separation in GelMA hydrogels was demonstrated. Combining HA and CS within a GelMA matrix had a synergistic effect, leading to molecular interactions and hydrogen bonding that resulted in several beneficial attributes, such as (i) less phase separation between HA and GelMA, (ii) enhanced mechanical properties compared to the addition of one GAG alone, (iii) tailorable rheological properties that infer printability of the hydrogels, and (iv) enhanced fibroblast proliferation and metabolic behaviour. These results show that these biomaterials have potential as microenvironments within a mechanical support to promote tissue regeneration.

## Figures and Tables

**Table 1:** Hydrogel formulations overview.

Name	GelMA	CS	HA
GelMA	10%		
GelMA/CS	10%	1%	
GelMA/HA	10%		0.5%
GelMA/CS/HA	10%	1%	0.5%

## Data Availability

Data are contained within the article.
